# Effects of online parietal transcranial electric stimulation on associative memory: a direct comparison between tDCS, theta tACS, and theta-oscillatory tDCS

**DOI:** 10.1038/s41598-022-18376-5

**Published:** 2022-08-18

**Authors:** Marko Živanović, Jovana Bjekić, Uroš Konstantinović, Saša R. Filipović

**Affiliations:** 1 Institute of Psychology & Laboratory for Research of Individual Differences, Faculty of Philosophy, University of Belgrade, Belgrade, Serbia; 2 Human Neuroscience Group, Institute for Medical Research, University of Belgrade, Belgrade, Serbia

**Keywords:** Cognitive neuroscience, Learning and memory, Neuroscience, Biological techniques, Electrophysiology

## Abstract

Associative memory (AM) is the ability to remember and retrieve multiple items bound together. Previous studies aiming to modulate AM by various transcranial electric stimulation (tES) techniques were inconclusive, although overall suggestive that tES could be a tool for AM enhancement. However, evidence from a direct comparison between different tES techniques is lacking. Here, in a sham-controlled cross-over experiment, we comparatively assessed the effects of three types of tES—anodal tDCS, theta-band transcranial alternating current stimulation (tACS), and theta-oscillatory tDCS (otDCS), delivered over the left posterior parietal cortex, during a short-term digit-color AM task with cued-recall. The effects were tested in 40 healthy young participants while both oscillatory tES were delivered at a previously determined individual theta frequency (4–8 Hz). All three active stimulations facilitated the overall AM performance, and no differences could be detected between them on direct comparison. However, unlike tDCS, the effects of which appeared to stem mainly from the facilitation of low-memory demand trials, both theta-modulated tACS and otDCS primarily promoted AM in high memory demand trials. Comparable yet differential effects of tDCS, theta tACS, and otDCS could be attributed to differences in their presumed modes of action.

## Introduction

Associative memory (AM) is the ability to form links between previously unrelated pieces of information so that the subsequent presentation of one activates the memory of the other. AM enables encoding and recall of the relationships between two or more items or between an item and its context, which makes it essential for everyday functioning. The decline of AM is seen in normal and pathological ageing^1^, and AM deficits are recognized as one of the most prominent early signs of mild cognitive impairment^2^ and neurological conditions such as dementia^3–5^. Therefore, the possibility of modulating AM using different non-invasive brain stimulation techniques such as transcranial magnetic stimulation (TMS) and transcranial electric stimulation (tES) has been gaining increased interest in recent years.

The advantages of tES in terms of safety, accessibility, flexibility, and cost^6^, led to the expansion of its research application in memory and other cognitive functions. tES is an umbrella term for several types of stimulation, including transcranial direct current stimulation (tDCS) and transcranial alternating current stimulation (tACS)^7^. Even though all tES techniques use low-intensity currents (1-2 mA) to modulate brain activity, they have different mechanisms of action due to the differences in the current waveform that is applied. Namely, tDCS modulates neuronal excitability via anodal/positive or cathodal/negative stimulation through depolarization or hyperpolarization of the resting membrane potential^8^. Unlike tDCS, which uses constant current flow between two electrodes of opposite polarity^9^, transcranial alternating current stimulation (tACS) applies the current that rhythmically switches polarity by oscillating at a specific frequency^10^. These rhythmic changes in current intensity lead to the membrane potential alternating between hyperpolarization and depolarization, which entrains endogenous neural oscillations to the tACS frequency^11^. Finally, oscillatory current stimulation or oscillating tDCS (otDCS)^12^ delivers frequency-modulated current as well, but without polarity shifts. Namely, the current oscillates in a sinusoidal manner at a specific frequency within either positive (e.g., between + 1 and + 2 mA) or negative polarity. Thus, anodal/positive otDCS is expected to increase excitability similar to tDCS, as well as to induce entrainment-like effects^13^.

The posterior parietal cortex (PPC) emerged as the most promising stimulation site following a series of MRI-guided TMS experiments^14–18^ showing AM enhancement through strengthening parieto-hippocampal functional connections. In addition, the tDCS studies focusing on AM showed positive effects of anodal stimulation over temporo-parietal^19^ and parietal cortex^20^, while stimulation over frontal regions resulted in mixed findings^21–23^. Generally, in tES studies investigating parietal stimulation and AM, the effects have been observed more systematically for cued recall^23–25^ (i.e., when participants are presented with one item from the pair and asked to recall the other) and when the stimulation is applied during the encoding^23,25,26^ (i.e., learning of the associated pairs). Moreover, due to the functional relevance of theta oscillations for AM^27^, the rhythmic stimulation protocols (tACS and otDCS) are usually delivered at theta-band frequencies (4–8 Hz) to induce memory-specific effects^28,29^, and it has been suggested that individually adjusted stimulation frequency will additionally improve tES effects^30,31^.

It is difficult to evaluate the effectiveness and/or differential effect on AM when tES techniques are applied in separate experiments. Namely, the heterogeneity of research designs, stimulation parameters (e.g., duration, intensity, and frequency of the stimulation, electrode position size, and shape), and memory outcome measures (type of paradigm, type and the modality of the stimuli, task difficulty) prevent direct comparison of the results. Moreover, only a few studies directly contrasted the effects of different tES techniques on AM. Namely, Lang and colleagues^32^ applied high-definition fusiform cortex stimulation (custom electrode montage: P10-Fp1-P2-P3-PO7) during encoding of face-scene pairs and found that 6 Hz-tACS but not 2 mA tDCS enhanced AM performance in healthy adults. Similarly, Klink et al.^33^ found that 20 min of 5 Hz-tACS (± 1 mA) but not tDCS (2 mA) applied over the ventrolateral prefrontal cortex during encoding improves AM in healthy older adults, but only when controlled for age. However, Vulić et al.^13^ showed facilitatory effects of both 5 Hz-otDCS (1.5 ± 0.1 mA) and anodal tDCS (1.5 mA) applied over the posterior parietal cortex (P3) for 20 min on face-word cued recall in a group of young adults. In contrast, Meng et al.^34^ found that 15 min 6 Hz-tACS (± 1 mA) applied over the posterior parietal cortex with a specific type of ring electrodes decreases AM of faces and scenes. Thus, it remains unclear how different tES techniques modulate AM and if/how their different modes of action translate to behavioral effects, i.e., AM performance.

In this study, we aimed to comparatively assess the effects of constant anodal tDCS, theta-band tACS, and theta-band otDCS on short-term AM. To enable direct comparison, we applied all stimulation types to the same group of participants, thus controlling for any potential differences that might stem from individual differences in neuroanatomy, neurophysiology, and cognitive abilities. Furthermore, we delivered stimulation over the left PPC using the same electrode set-up across all conditions. In addition, this is the first study to apply frequency-personalized tACS and otDCS to modulate AM by applying the stimulation at individual theta frequency extracted from EEG data during successful associative encoding^35^. Finally, we considered different outcome measures to explore the specificity of the effects. We hypothesized that all active tES promote AM performance, and we explored if tDCS, tACS, and otDCS have differential cognitive effects that can be attributed to their assumed modes of action.

## Methods

### Experimental design

The study adopted a fully-repeated, cross-over, sham-controlled design in which each participant received constant anodal tDCS, tACS, otDCS, and sham stimulation in counterbalanced order while they performed parallel forms of cognitive tasks. Thus, the experiment was designed to assess the cognitive effects during tES in the so-called *online* protocol.

### Participants

Forty healthy volunteers 22–35 years of age (25.15 ± 3.66 years, 25 females) completed the experiment. Based on the a priori power analysis in G*Power^36^, the minimal sample size of 34 participants was required to detect medium effects size of *d* = 0.50^37^ with the power of 0.80, at the alpha level of 0.05 under the assumption of *r* = 0.50 between repeated measures.

All the participants were right-handed, native speakers with a normal or corrected-to-normal vision, and satisfying standard tES inclusion criteria^38^—none reported a history of psychiatric, neurological, acute, or chronic skin conditions, nor the use of psychoactive substances and medication. Participants provided informed consent and were compensated for their participation in the study (8€/h). The study was conducted in accordance with the Declaration of Helsinki and was approved by the Ethics Board of the Institute for Medical Research (protocol number EO129/2020).

### Transcranial electric stimulation (tES)

**Figure 1:**
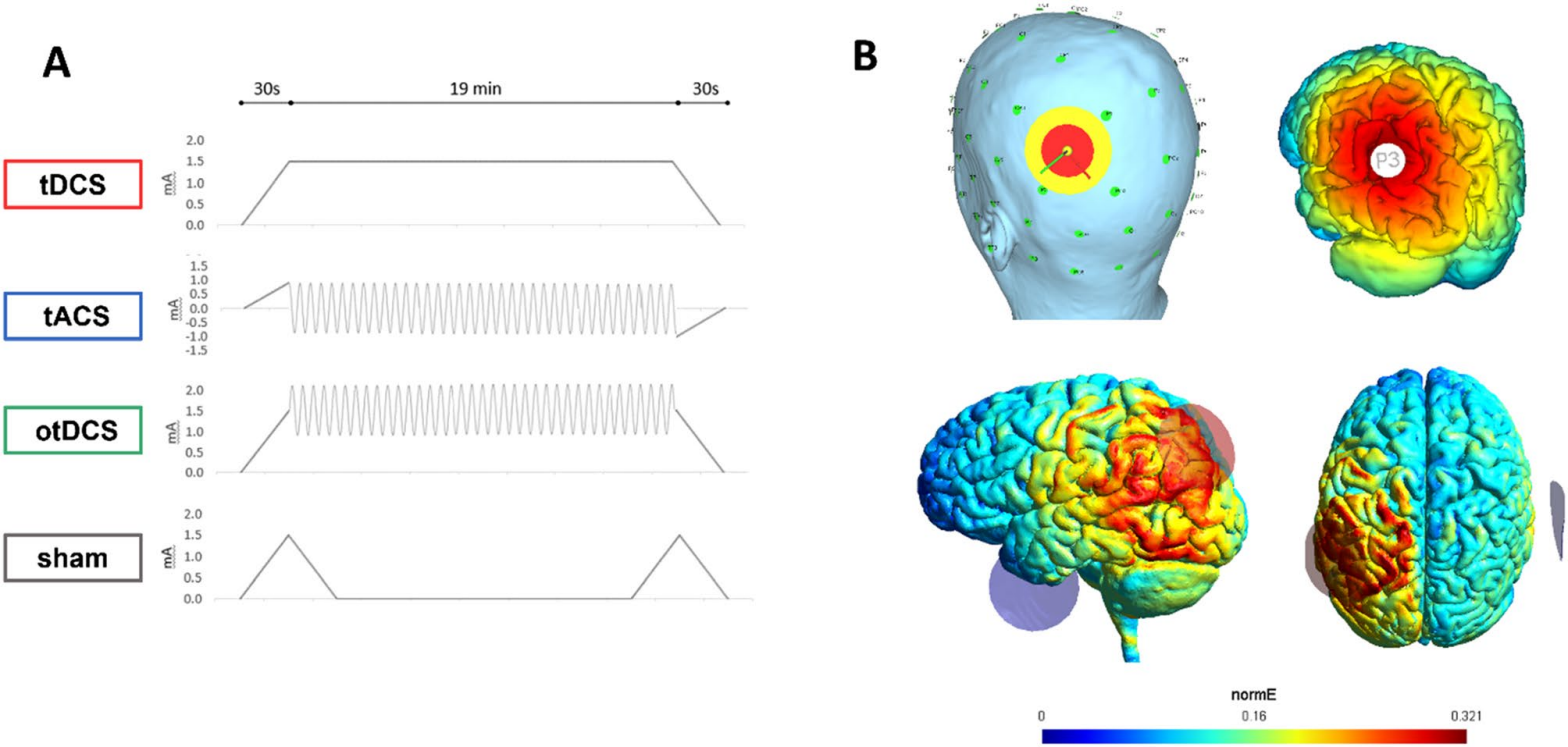
The transcranial electric stimulation: (**A**) anodal tDCS, tACS, otDCS, and sham protocols; (**B**) The left PPC electrode montage and the simulation of the electric fields in SimNIBS 3.2.6^41^. The simulation was built using the male head model and the following stimulation parameters: two round-shape 1 mm thick rubber electrodes in saline-soaked sponge pockets with a 25 cm^2^ contact surface; one electrode is placed at P3 and the other on the right cheek slightly below and anterior from the external auditory meatus. The images show an electric field generated by + 1.5 mA at P3 and—1.5 mA at the contralateral cheek, which represents the field during tDCS, as well as the average field during otDCS. The field induced by tACS is of slightly lower intensity (± 1 mA) and switches between polarities in theta rhythm (see Bjekić et al.^40^ for video of current field changes in time).

All stimulation protocols and procedures followed safety guidelines for the use of tES in human studies^39^. Stimulation was applied with a light, battery-operated hybrid tDCS-EEG Starstim device (Neuroelectrics Inc., Barcelona) attached to the back of the neoprene cap and remotely operated via Neuroelectrics® Instrument Controller (NIC2) software (Neuroelectrics Inc., Barcelona, Spain). Across all conditions, tES was delivered via two round rubber electrodes inserted in saline-soaked sponge pockets and placed on participants' heads. The contact surface of both electrodes was 25cm^2^^35^, and approximately 10 ml of saline was used per sponge^40^. To stimulate the left PPC, one electrode was positioned over P3 of the 10–20 International EEG system, while the return electrode was placed on the contralateral cheek. The PPC emerged as the most promising stimulation target for AM modulation in a series of MRI-guided TMS studies^15–18^. The return electrode was placed on the contralateral cheek to avoid inducing physiologically relevant changes to function-unrelated cortical areas (Fig. 1). The P3-contralateral cheek electrode montage was successfully used in previous tES studies targeting AM^13,20,24^.

In the anodal tDCS condition, the constant current was delivered at 1.5 mA (Fig. 1). The tACS was delivered at ± 1 mA (i.e., 2 mA peak-to-peak) intensity and previously determined individual theta frequency (ITF). The same intensity was applied in previous studies to minimize retinal phosphenes^33^. The otDCS was delivered with the intensity of the current sinusoidally fluctuating ± 0.5 mA around 1.5 mA (i.e., between 1 and 2 mA), also at ITF. The ITF was determined for each participant in a pre-experiment EEG session, where they completed a typical (i.e., face-scene) AM task while EEG was recorded^42^. As a result, the tACS/otDCS were delivered in frequencies between 4 and 7.5 Hz (*M* = 5.18 Hz, *SD* = 1.17 Hz). We can assume the equivalence between tDCS and otDCS protocols in terms of current intensity (1.5 mA on average) and the comparability of oscillating properties between tACS and otDCS since both have been delivered at ITF. Finally, in the sham condition, the current was delivered during the first and last 60 s (30 s ramp up/down) to emulate the sensations at the beginning/end of the real stimulation.

In all stimulation conditions, the current was applied for 20 min with 30 s ramp-up/down periods at the beginning and end. The impedance was checked before and during tES to assure that it was always below 5 kΩ.

### The subjective experience of tES

To evaluate the tolerability of different tES types, the participants were asked to report the level of discomfort on a 10-point scale (1—a complete absence of any unpleasant sensations, 10—extreme unpleasantness) during stimulation, i.e., after the first minute of stimulation onset and after each task was completed (approx. minutes 8 and 16). In addition, participants were asked to report any adverse effects or unusual sensations. The potential adverse effects were assessed by a self-report questionnaire, which consisted of 13 possible side effects (headache, neck pain, back pain, blurred vision, skin irritation, prickling/tingling sensation, itching, increased heart rate, burning sensation, dizziness, acute mood swings, tiredness, anxiety).

### Cognitive tasks

The tasks were programmed in OpenSesame^43^ and presented on a 23′′ monitor (resolution: 1920 × 1080, refresh rate 60 Hz).

#### Short-term associative memory task

**Figure 2:**
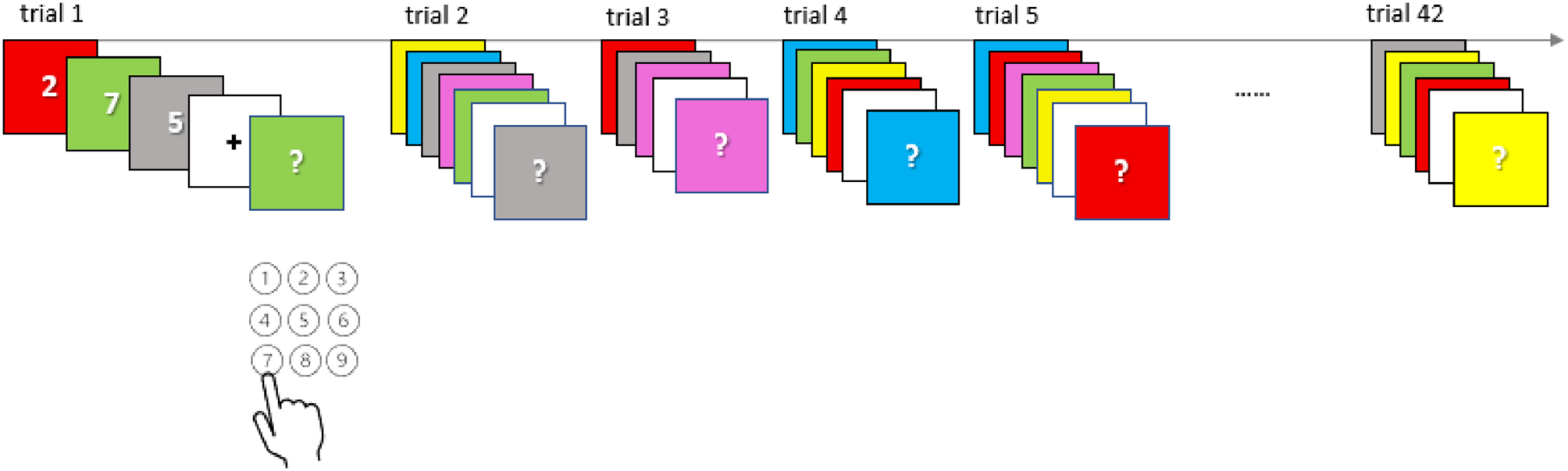
Short-term associative memory task scheme.

The short-term associative memory (stAM) task consisted of 10 single digits (0 to 9) printed in white ink and presented sequentially on a different color cards (red, blue, green, yellow, gray, or pink) in a fixed interval of 1250 ms with the interstimulus interval (ISI) of 250 ms. Participants were instructed to remember each digit-color association in the sequence. At the end of each sequence (signalized by the appearance of the 1250 ms fixation point), the participants were presented with a cue, i.e., one of the previously seen color cards, and needed to recall the digit shown on a given color (Fig. 2). The length of digit-color sequences varied between 3 (low-demand trials) to 5 stimuli (high-demand trials). The target digit could appear at each position in a sequence, and the frequency of these presentations was balanced across sequences. In total, 42 sequences were successively presented in fixed pre-randomized order.

#### Control task

To determine the specificity of tES effects, the Simon task was included as a control measure. In this task, words “left” and “right” are sequentially presented on the screen at (635px either left or right of the center). The participant's task was to respond as fast as possible by pressing either the left or right button in correspondence to the word's meaning, regardless of the position on the screen. There were 150 trials in total (75 right/75 left), and in each trial, the stimulus was preceded by a 300 ms fixation point, while a 500 ms white screen followed each response.

### Procedure

The experiment consisted of four tES sessions, one for each type of stimulation—tDCS, tACS, otDCS, and sham. The order of the conditions was counterbalanced across participants. For each session, participants came to the laboratory at the same time of day to minimize the impact of the circadian rhythm and vigilance fluctuations as a potential confounding factor^44,45^. Experimental sessions were at least 1 week apart to prevent potential carry-over effects^40^.

At the beginning of each session, participants reported their current mood (1—extremely bad mood, 10—extremely good mood) and level of tiredness (1—extremely tired, 10—very well rested). The side-effects self-report questionnaire was administered before and immediately after tES.

The cognitive tasks were administered during tES in a fixed order. Namely, the stAM task started 3–4 min after the stimulation protocol onset, while the control task was administered approximately 11 min after the beginning of the stimulation. Parallel forms of the task were used to minimize the possible confounding effect of learning, and their order was counterbalanced across participants and stimulation conditions.

Following the last experimental session, all participants were debriefed in detail. To assess the successfulness of blinding, the end-of-study-guess procedure was adopted—that is, after the experiment, participants were asked to guess in which of the previous sessions they received sham stimulation.

### Data analysis

The primary outcome measure from the stAM task was the short-term associative cued recall accuracy, i.e., the total number of correctly cue-recalled digits (transformed to percentages). In addition, two sub-scores depending on the sequence difficulty were calculated, i.e., cued recall accuracy for the 3-item (i.e., low-demand) and the 5-item (i.e., high-demand) sequences. As a secondary measure, we calculated recall time, i.e., the average time between cue onset (color card) and the response for correct trials. As a control measure, we used the average reaction time from the Simon task.

Extreme or outlier recall times in the stAM task, as well as reaction times in the Simon task, were identified as ± 3*SD* first between subjects and then within each subject, per condition. Trimming (i.e., replacement with upper and lower bound of the ± 3*SD* interval) was done separately for each sequence length and type of stimuli in the Simon task. Finally, all data were inspected for extreme underperformance, i.e., scores on cognitive tasks outside the individual's 95% performance range in respect to average group-level variability were replaced with individual sham-control performance (2.1% of replacements were made in total).

Data analyses were performed in JASP 0.14.1^46^, and descriptive statistics (*M* and *SD*) were calculated for all variables. The effects of stimulation were assessed using the repeated measure analysis of variance (ANOVA) with simple within-subjects planned contrasts enabling comparisons of each active stimulation to the sham condition (tDCS vs. sham, tACS vs. sham, and otDCS vs. sham). When Mauchly’s W statistic was non-significant, the sphericity was assumed, otherwise, the correction for unequal variances was applied. To compare the performances between three active conditions, Holm-corrected *post-hoc* tests were used. We report effect sizes (Cohen’s *d*) alongside the exact alpha level for statistical significance.

For the relationship between stimulation-induced changes in AM and the differences in mood/tiredness, we employed a correlational approach (Pearson *r*). Finally, a formal assessment of blinding was performed using a Binomial test with a priori probability of 0.25 (H_1:_ correct guess proportion > 0.25).

## Results

### Participants’ state, tES tolerability, and adverse effects

The stimulation was well tolerated with the average reported levels of discomfort below 2.5 on 1 to 10 scale across all conditions: tDCS 2.43 (*SD* = 1.24), tACS 2.00 (*SD* = 1.11), otDCS 2.41 (*SD* = 0.96), and sham 1.62 (*SD* = 0.58). Nevertheless, all active tES were rated more unpleasant than sham—tDCS [*t*_(39)_ = 4.917, *p* < 0.001, *d* = 0.717], tACS [*t*_(39)_ = 2.202, *p* = 0.034, *d* = 0.336], and otDCS [*t*_(39)_ = 5.850, *p* < 0.001, *d* = 0.702]. Anodal tDCS and otDCS did not differ in respect to unpleasantness (*p* = 0.923), while tACS showed trend-level less discomfort than both anodal tDCS (*p* = 0.071, *d* = 0.380) and otDCS (*p* = 0.071, *d* = 0.365).

Participants did not show differences in reported tiredness across conditions: sham versus tDCS [*t*_(117)_ = 0.371, *p* = 0.711], sham versus tACS [*t*_(117)_ = 0.965, *p* = 0.337], sham versus otDCS [*t*_(117)_ = 0.371, *p* = 0.711], as well as no differences in mood: sham versus tDCS [*t*_(117)_ = 0.339, *p* = 0.735], sham versus tACS [*t*_(117)_ = 0.932, *p* = 0.353], sham versus otDCS [*t*_(117)_ = 1.017, *p* = 0.311].

On the individual level, phosphenes were reported during theta otDCS by one participant. During tACS, seven participants reported blurred vision, and three of them reported phosphenes too. On the group level, tACS was rated the most comfortable by 45% of participants, tDCS by 15% of participants, and otDCS by 12.5% of participants.

### Effects on AM performance

The associative cued recall accuracy i.e., the percentage of correctly recalled digit-color pairs, was 66% during sham (*range* = 42.9–100%), 70% during constant anodal tDCS (*range* = 47.6–97.6%), 70% during tACS (*range* = 42.9–95.2%) and 69% during theta otDCS (*range* = 38.1–95.2%). Descriptive statistics for all measures are presented in Table 1.

**Table 1:** Descriptive statistics (mean and standard deviation) during sham, anodal tDCS, tACS, and otDCS conditions for accuracy (%) in the short-term AM task (overall performance, low- and high-demand sequences), stAM recall time (ms), and the control task reaction time (ms).

	Sham	tDCS	tACS	otDCS
	M ± SD	M ± SD	M ± SD	M ± SD
stAM overall accuracy	66.31 ± 15.23	69.88 ± 14.02	70.18 ± 14.64	69.40 ± 16.17
stAM low-demand	76.61 ± 17.12	82.50 ± 14.10	80.89 ± 17.38	79.11 ± 18.68
stAM high-demand	52.86 ± 18.21	58.39 ± 18.65	59.11 ± 17.19	58.93 ± 19.53
stAM RT	1645.55 ± 323.92	1645.62 ± 310.84	1620.41 ± 281.77	1643.67 ± 300.44
Control task	578.06 ± 52.39	583.41 ± 47.81	578.10 ± 54.79	584.78 ± 50.23

**Figure 3:**
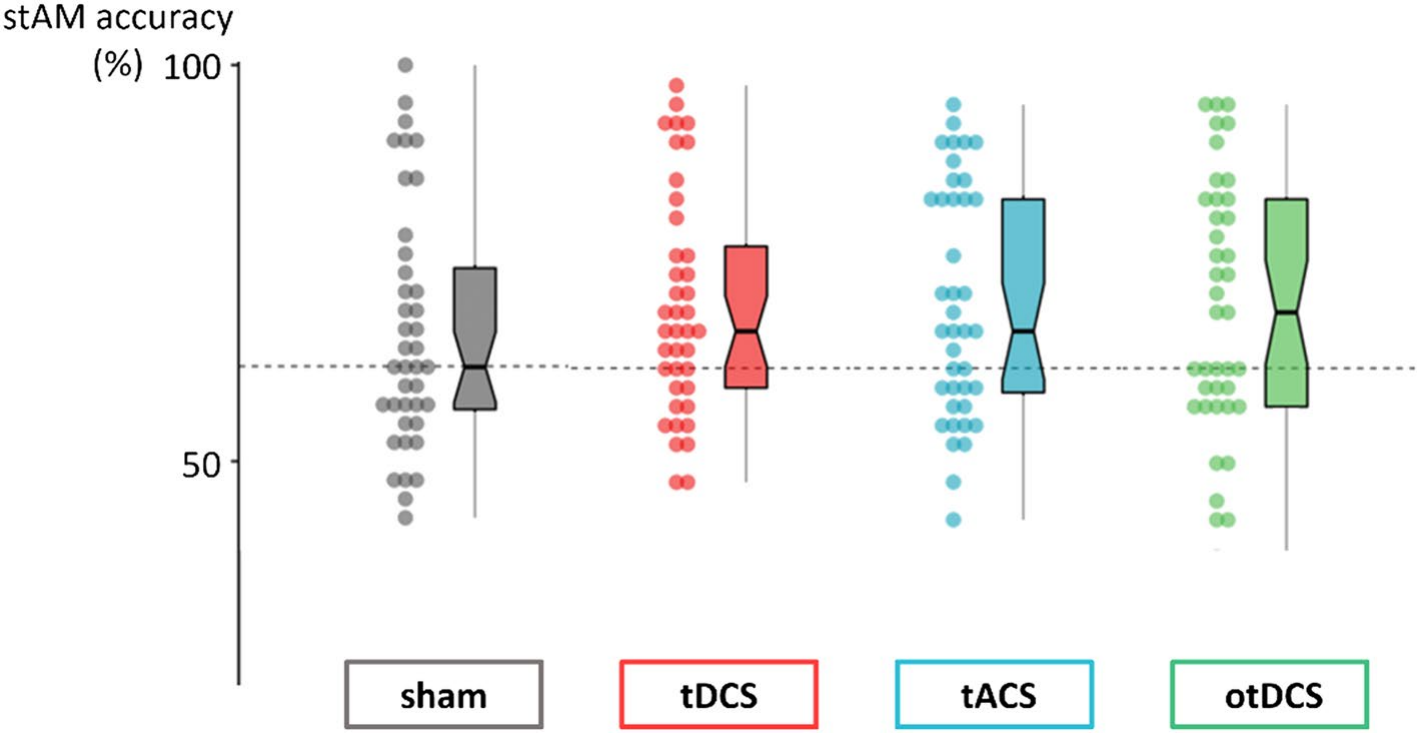
The short-term AM accuracy (%) during sham, tDCS, tACS and otDCS. Individual data points are presented alongside boxplots showing the median and interquartile range.

The repeated measures ANOVA planned sham-contrasts showed that theta-frequency tACS clearly increased short-term AM accuracy [*t*_(117)_ = 2.342, *p* = 0.021, *d* = 0.370]. Solid facilitatory effect was also observed for constant anodal tDCS [*t*_(117)_ = 2.162, *p* = 0.033, *d* = 0.342], while the improvement during theta otDCS was recorded at the trend-level [*t*_(117)_ = 1.873, *p* = 0.064, *d* = 0.296]. The *post-hoc* comparisons between each of the active tES conditions showed no significant differences in short-term AM accuracy (*p*-values for all comparisons = 1.00). The results are presented in Fig. 3.

To account for ceiling effects, the analysis was repeated without participants whose average performance across all conditions was > 90% (*n* = 4). Now, the sham-contrasts showed clearly better average performance during all three tES conditions—tACS [*t*_(105)_ = 2.695, *p* = 0.008, *d* = 0.449], tDCS [*t*_(105)_ = 2.252, *p* = 0.026, *d* = 0.375], and otDCS [*t*_(105)_ = 2.031, *p* = 0.045, *d* = 0.338]. Same as for the full set of participants, no differences between active tES conditions could be seen (all *p*-values = 1.00).

As the position of the target digit varied within the sequence, the recency effect^47^ (i.e., higher recall success when the target digit was at the last position) was checked and was found to be relatively high (88% success for last-position vs. 62% for all other positions). To account for this, we recalculated the scores on the stAM task without trials in which the target digit was at the last position. The tACS retained its effect which was almost the same as when the full set of trials was analyzed [*t*_(117)_ = 2.150, *p* = 0.034, *d* = 0.340], the otDCS effect became slightly stronger reaching the edge of significance [*t*_(117)_ = 1.944, *p* = 0.054, *d* = 0.307], while tDCS effect diminished considerably [*t*_(117)_ = 1.696, *p* = 0.093, *d* = 0.268]. Still, no statistical differences were found between any of the active tES conditions (all *p*-values = 1.00).

Significant differences in difficulties between low- and high-demand sequences were obtained [*F*_(1,39)_ = 192.756, *p* < 0.001], thus we assessed tES effects depending on the cognitive demand. We found that the performance on low-demand sequences was improved during tDCS [*t*_(117)_ = 2.556, *p* = 0.012, *d* = 0.404] with trend-level effect during tACS [*t*_(117)_ = 1.859, *p* = 0.066, *d* = 0.294], while otDCS did not show any effect [*t*_(117)_ = 1.084, *p* = 0.260, *d* = 0.171]. In contrast, performance on high-demand sequences was improved by tACS [*t*_(117)_ = 2.138, *p* = 0.035, *d* = 0.338] and otDCS [*t*_(117)_ = 2.077, *p* = 0.040, *d* = 0.328], while tDCS showed only trend-level effect [*t*_(117)_ = 1.894, *p* = 0.061, *d* = 0.299].

With the recency effect removed (i.e., without the last-position trials), in low-demand sequences, an altered pattern of effects was seen—tDCS effect turned out to be at trend-level [*t*_(117)_ = 1.879, *p* = 0.063, *d* = 0.297], and was similar to the otDCS effect [*t*_(117)_ = 1.793, *p* = 0.075, *d* = 0.284], while the effect of tACS could not be detected [*t*_(117)_ = 1.196, *p* = 0.234, *d* = 0.189]. However, in high demand sequences, a convincing tACS effect was observed [*t*_(117)_ = 2.666 *p* = 0.009 *d* = 0.422], as well as otDCS effect [*t*_(117)_ = 2.148, *p* = 0.034, *d* = 0.340], while anodal tDCS effect remained at the trend level [*t*_(117)_ = 1.852, *p* = 0.067, *d* = 0.293], which was all not much different than what had been seen before removal of the recency effect.

Finally, no effects on AM recall times were observed in the stAM task. Namely, the time to recall the correct digit when presented with the cue was no different during anodal tDCS [*t*_(117)_ = 0.002, *p* = 0.999], tACS [*t*_(117)_ = 0.623, *p* = 0.534], and otDCS [*t*_(117)_ = 0.047, *p* = 0.963] in comparison to the sham condition. However, it is important to note that the recall times were rather long (between 1 and 2 s) with high interindividual variability, as the responses were not time constrained.

### Potential confounding factors and the effects on the control task

We ran additional analyses to exclude the possibility that the obtained effects on AM are accidental effects of other confounding factors. First, we assessed if the differences in AM accuracy can be explained by differences in sensations induced by different tES conditions, as well as self-reported mood and tiredness. To test this, we correlated differential scores for AM accuracy between each active stimulation and sham with the corresponding differential scores for the level of discomfort, self-reported mood, and tiredness. We found no significant correlation between stimulation-induced discomfort relative to sham and corresponding improvement in AM performance in anodal tDCS (*r* = − 0.095, *p* = 0.558), tACS (*r* = 0.083, *p* = 0.613), nor otDCS (*r* = − 0.091, *p* = 0.577). Furthermore, we found no correlation between the changes in AM and differences in participants’ self-reported mood (anodal tDCS *r* = 0.071, *p* = 0.665; tACS *r* = 0.021, *p* = 0.895; otDCS *r* = 0.021, *p* = 0.899), or levels of tiredness (anodal tDCS *r* = − 0.095, *p* = 0.558; tACS *r* = 0.083, *p* = 0.613; otDCS *r* = − 0.091, *p* = 0.577).

To assess the potentially confounding effect of cross-session practice, we compared AM performance based on session order regardless of the stimulation condition. The results showed that participants had lower AM accuracy in the first session in comparison to each of the subsequent sessions (S1 vs. S2: *p* = 0.022, S1 vs. S3 *p* < 0.001, and S1 vs. S4 *p* < 0.001), while no differences between sessions 2, 3 and 4 were observed (*p*-values: 0.233–0.699). To exclude the possibility that the practice effects were confounded with stimulation effects, the main analysis was repeated, but this time with AM accuracy scores that were centered to the order of session in which they were recorded. The results remained unchanged—namely, tACS and constant anodal tDCS increased short-term AM accuracy [*t*(117) = 2.442, *p* = 0.016, *d* = 0.386; *t*(117) = 2.314, *p* = 0.022, *d* = 0.366, respectively], while the improvement during theta otDCS was observed at the trend-level [t(117) = 1.840, *p* = 0.068, d = 0.291]. This was also true for accuracy on low-demand sequences which was improved only during tDCS [*t*_(117)_ = 2.600, *p* = 0.011, *d* = 0.411] and performance on high demand sequences that was facilitated by both tACS [*t*_(117)_ = 2.146, *p* = 0.034, *d* = 0.339] and otDCS [*t*_(117)_ = 2.008, *p* = 0.047, *d* = 0.318].

In addition, the results showed that the end-of-the-study sham-guess was at the chance level (Binomial test *p* = 0.560), indicating adequate participants' blinding at the group level. However, since 10 participants (25%) correctly guessed sham condition, we assessed the effect of guessing as a between-subject factor and found that it did not affect AM performance [*F*_(1,38)_ = 0.460, *p* = 0.502], nor was there significant guessing x stimulation interaction effect [*F*_(3,114)_ = 0.661, *p* = 0.578].

Finally, we analyzed the stimulation effects on the control task, and found no differences between sham and any of tES conditions—anodal tDCS [*t*_(117)_ = 1.022, *p* = 0.309], tACS [*t*_(117)_ = 0.007, *p* = 0.994] and otDCS [*t*_(117)_ = 1.283, *p* = 0.202], as well as no differences between the three active stimulation conditions (all *p*-values = 1.00).

## Discussion

The study comparatively assessed the effects of three types of tES on short-term AM. This is the first study that directly compared the effects of constant anodal tDCS, tACS oscillating in theta rhythm, and theta oscillatory-modulated anodal tDCS (i.e., otDCS). The results generally show that all three stimulation types can improve concomitant short-term AM performance. However, in keeping with their different presumed modes of action, the three tES techniques also showed some peculiarities in their effects.

The well-established role of theta rhythm in memory^27,48^ led to the development of theta-frequency modulated tES protocols. The idea behind theta-frequency tACS is that the rhythmical externally applied current oscillations in theta frequency will entrain the intrinsic oscillatory activity of the wide-range functional brain networks^49,50^, promoting theta synchronization^51^, and as a result, enhance memory performance^32^. The effects of tACS observed in this experiment are in concert with previous studies showing improved AM during theta-tACS^32,33^. Interestingly, a recent study that also applied theta-tACS over the left posterior parietal cortex found an AM-specific effect in the opposite direction^34^. The authors put forward a hypothesis that lower AM performance in their experiment could be explained by the frequency of the stimulation, which was set at 6 Hz, which might have happened to be different from the endogenous theta frequencies of most of the participants and consequently led to shifts in phase and/or drops in synchronization of the endogenous frequencies, thus resulting in net disruption of memory processing^34^. To avoid this potential issue, in our study, we did not use a fixed stimulation frequency for all participants but the individual theta frequency extracted from EEG during an AM task. However, given that several previous studies reported positive effects during fixed-frequency tACS on different cognitive outcomes^29^, it is difficult to decide whether stimulation at the individual theta frequency is really necessary to enhance AM performance.

Two previous studies that compared tACS and constant anodal tDCS stimulation found tACS but not tDCS to be effective in modulating concurrent AM performance^32,33^. Here, we also observed the effect of tACS, but the positive effect of constant anodal tDCS was found too. The discrepancy may be due to the fact that in contrast to our study in which PPC was targeted, one of the previous studies targeted the fusiform cortex to enhance the encoding of face-picture associations^32^, while the other stimulated the left ventrolateral prefrontal cortex^33^. This would be in keeping with the conclusion of the meta-analysis by Galli et al.^21^ that the tDCS effects on memory are stimulation-site dependent, with the effects for left parietal stimulation to be higher than the ones reported following the stimulation of the other brain loci. In support of this, several TMS studies showed that the posterior parietal cortex is quite an effective target for AM enhancement^14,15,17,52^, while our previous studies with anodal tDCS delivered before various AM tasks consistently showed beneficial effects of parietal stimulation^13,20,24^.

The novel otDCS approach has been designed with the idea of bringing together the benefits of both tDCS and tACS in a single technique. The sinusoidal oscillations superimposed on an anodal DC potential are supposed to be able to induce both neuroplasticity changes of the standard anodal tDCS (i.e., increased excitability), as well as the tACS-like entrainment of the endogenous brain oscillations (due to the rhythmic fluctuations of the membranes' potential). Hence, theta otDCS should be able to produce comparable if not even more pronounced effects than tDCS/tACS. In this study, we did observe a positive otDCS effect, but it was of a less convincing magnitude than expected. In fact, theta otDCS induced AM improvement was clearly shown only when top-performing individuals were excluded. This puts forward the importance of task difficulty and the level of performance that may act as a limiting factor for observing the effects of non-invasive neuromodulation techniques on cognition, in this case, the effects of otDCS. Nevertheless, it needs to be highlighted that this is the first conceptual replication of the previously reported beneficial effect of theta otDCS on face-word associations^13^. The main difference is that the effects here are observed during the stimulation, as opposed to after the stimulation in the previous study, suggesting thus the ability of the technique to induce changes in ongoing brain activity and related task performance. Another relevant point to stress is that the otDCS effects have now been observed in diverse types of task/stimuli (i.e., face-word cued recall in the previous study^13^ vs*.* short-term color-digit recall in this study), thus providing evidence that the ability of theta-modulated otDCS to facilitate AM performance is not task- or stimuli-specific. Further, more detailed neurophysiological studies are needed to untangle precise mechanisms behind the otDCS effects.

The direct comparison between the anodal tDCS, tACS, and otDCS did not show a differential effect on overall AM performance. Namely, all three types of stimulation produced small-to-medium effect sizes (Cohen *d* 0.30 – 0.45), with tACS seemingly inducing slightly stronger effects. However, the differences between tDCS, tACS, and otDCS were too small to be detected and thus could be considered as probably negligible, even if statistically significant, in the context of the comparative advantage of one technique over the other. Despite relying on distinct physiological mechanisms, it seems that all three techniques have similar behavioral net effects, at least when the short-term AM is concerned.

However, a closer look at the AM facilitation patterns revealed differential effects of tDCS, tACS, and otDCS that appeared to be dependent on the level of memory demand. When the distinction between low- and high-demand sequences was analyzed, anodal tDCS showed to be much more effective in improving performance on low-demand than on high-demand sequences, while theta-frequency oscillatory tES (tACS and otDCS) effects were observed primarily on high-demand sequences. This was further confirmed by the change in the overall effectiveness of the three tES techniques introduced by removing the recency effect, which effectively removed the easiest trials from the analysis. Namely, with the recency effect removed, the effects of tACS and otDCS remained the same or even improved, suggesting that their main effects were not much dependent on improvement in trials with the lowest memory demand. In contrast, the effect of tDCS dropped well below the significance level suggesting that a considerable part of its effect was due to improvement in trials with the lowest memory demand. It is unlikely that differential effects could be attributed to the differences in stimulation parameters such as current intensity (tDCS and otDCS show different effects despite equivalent intensity) and/or oscillation amplitude (tACS and otDCS show similar effects despite different amplitudes).

The tES effects have been reported to depend on performance level^53^, however previous studies assessed the differences between high- and low-performing individuals, not between different levels of task difficulties in the same group of people. The differences between high- and low-demand sequences could be just a chance-finding, but it could also provide a window into underlying mechanisms of the tES effects on short-term AM. This way, the results could imply that anodal tDCS-induced enhancement is mediated by the facilitation of lower-level memory processes, more salient in low-memory demand than in high-memory demand situations, while theta oscillatory tES (tACS and otDCS) seem to facilitate performance that requires more efficient encoding and/or longer retention. At the physiological level, the tDCS effects on AM performance probably result mostly from increased excitability of the neural circuits below the active electrode, while the tACS/otDCS effects would be more likely to be caused by modulation of the long-range communications in the cortico-hippocampal network through entrainment of theta activity which in turn promotes associative binding. However, the evaluation of this hypothesis would need a specifically designed study with a more detailed assessment of associative encoding and retention at different levels of memory demands.

Furthermore, it is of note that the novel theta otDCS technique tended to produce effects that followed our initial assumptions regarding its mode of action. This was particularly clear when low- and high-demand sequences were compared with the recency effect removed. In low-demand sequences, otDCS and tDCS were of similar magnitude, while tACS effects were not detectable. On the other hand, in high-demand sequences, tACS and otDCS were clearly more effective in contrast to sham than tDCS was. In other words, the pattern of otDCS effects appears as an additive mix of tDCS and tACS effects.

Another factor to be considered is the personalized frequency (ITF) that was used to tailor oscillatory protocols (tACS/otDCS) by matching them with EEG theta rhythm recorded during successful memory encoding. This was done to maximize the resonance effect, i.e., to amplify a network's activity by stimulation at its function-relevant “natural” frequency. However, since this study did not assess individualized versus non-individualized protocols, the relative contribution of frequency-personalization to the effectiveness of oscillatory stimulation techniques and their effects on different memory demands needs to be further explored.

Nevertheless, regardless of the particularities of the three tES techniques, our data convincingly show that the observed effects are specific to memory processes, as no effects have been observed on the control task, or even recall speed measure obtained from the same stAM task. This is in line with previous experiments showing function-specific effects of parietal stimulation on AM^13,20^.

Since this is the first study to assess anodal tDCS, tACS, and otDCS in the same group of participants, it is important to stress that all stimulation techniques were well tolerated (i.e., rated 2.5 or less on a 1–10 scale), despite all being rated slightly more uncomfortable than sham stimulation. The individual differences in reported adverse effects suggest that some participants are more sensitive to changes in current flow (e.g., 2 mA peak-to-peak in tACS), while others might be more sensitive to the intensity of the stimulation (1.5 mA in tDCS). Common tACS side effects such as blurred vision and phosphenes were also recorded in this study. Due to the placement of the return electrode, we believe the phosphenes induced are retinal rather than cortical. The variability in these sensations could be due to many reasons—but the simplest one might be individual differences in head anatomy. Namely, the return electrode was the same size for all participants, despite their different head/face sizes. As a result, the electrode might have been closer/further from the eye, and the current density in the eye might have varied between participants. Additionally, participants might have different thresholds for inducing phosphenes. Nevertheless, phosphenes were reported by a smaller number of participants compared to some other studies (17% vs. 47%^32^), probably due to differences in electrode montage. Interestingly, these differences in sensations/side effects did not compromise group-level blinding, which was at the chance level, and more importantly, did not affect performance. Still, it is important to note that theta otDCS induced visual adverse effects in only one participant as opposed to seven participants who experienced it during tACS.

Given the significance of memory decline in aging as well as the core role of memory impairments in Alzheimer’s and other dementias, developing approaches that could improve memory functions and ultimately prevent or even reverse memory loss is of utmost importance. Various tES techniques, if proved to be effective, due to their noninvasiveness, relatively low cost, and ease of application (with potential for home use) are particularly well suited for that purpose. Unfortunately, clinical studies exploring the effectiveness of tES are still scarce. So far, only tDCS has been investigated in clinical studies. However, a few recent systematic reviews on the effectiveness of tDCS in dementia concluded that although there was an overall beneficial effect of tDCS on cognition, due to the small number of studies and the high heterogeneity of the data, more high-quality studies using standardized parameters and measures are needed^54,55^. One of the main reasons is the lack of detailed knowledge regarding not only which tES technique is optimal for memory enhancement in various age groups (as mentioned in the Introduction), but also the lack of understanding of the specifics of the simulation application. The latter include, among other issues: areas to be stimulated and how (i.e., the electrode positioning), the intensity of stimulation, and, in the case of oscillatory protocols, the stimulation frequency. Our study aimed to provide just one stone in the mosaic of knowledge required to allow for the translation of basic research into clinical practice. The most important outcome is that it provides evidence that oscillatory protocols may have more function-specific effects, and therefore they appear as a promising path to pursue for future studies.

Before conclusion, several limitations of the current study need to be acknowledged. First, the participants were healthy young adults whose memory performance was at its optimal level. As a result, this was a high-performing sample, which might have acted as a limiting factor for the magnitude of tES effects to be observed. The conceptual replication of these findings in lower-performing groups would probably provide a better estimate of the effect sizes and potential differences in effectiveness across these three types of techniques, especially because low performers tend to benefit more from tES^24,53^. Furthermore, the AM assessment was less extensive than what would be required for drawing confident conclusions on differential effects on low and high-demand memory processes. To address that, future studies should aim at an increased number of trials at each level of memory demand and introduce higher memory load items (e.g., six- or even seven-digit sequences), thus additionally challenging the tES effects in high-performing individuals. In addition, it remains to be assessed if the same configuration of the effects would be obtained if a recognition rather than recall task is used, as well as if the effects observed during tES would sustain after the stimulation. Finally, in this study, we observed lower performance in the first session compared to subsequent sessions. Even though the tES effects were unaffected by this, it is advisable to include practice/familiarization sessions in future studies with cross-over designs, thus minimizing the risk of confounding the stimulation and practice effects.

## Conclusion

The application of different types of tES—anodal tDCS, theta-band tACS, and theta-modulated oscillatory stimulation (otDCS) over the parietal cortex showed facilitatory, function-specific online effects on short-term AM performance. The effectiveness of different techniques was comparable, but the effects varied depending on the task demand, with tDCS being more beneficial when the memory demand is relatively low, while theta-modulated tACS and otDCS proved to predominantly promote AM when the memory load is rather high. Overall, in the light of comparable effectiveness, it might be important to consider the level of memory demand, as well as subjective experience, i.e., tolerability of the procedure when making judgments about the applicability of different tES types in clinical and more vulnerable populations.

## Data Availability

The dataset generated during the current study is available from the corresponding author on reasonable request.
